# Kelp forests at the end of the earth: 45 years later

**DOI:** 10.1371/journal.pone.0229259

**Published:** 2020-03-11

**Authors:** Alan M. Friedlander, Enric Ballesteros, Tom W. Bell, Jennifer E. Caselle, Claudio Campagna, Whitney Goodell, Mathias Hüne, Alex Muñoz, Pelayo Salinas-de-León, Enric Sala, Paul K. Dayton

**Affiliations:** 1 Pristine Seas, National Geographic Society, Washington, DC, United States of America; 2 Hawai‘i Institute of Marine Biology, Kāneʻohe, Hawai‘i, United States of America; 3 Centre d'Estudis Avançats (CEAB-CSIC), Blanes, Spain; 4 Earth Research Institute, University of California Santa Barbara, Santa Barbara, California, United States of America; 5 Marine Science Institute, University of California Santa Barbara, Santa Barbara, California, United States of America; 6 Wildlife Conservation Society, Buenos Aires, Argentina; 7 Fundación Ictiológica, Santiago, Chile; 8 Charles Darwin Research Station, Puerto Ayora, Galápagos Islands, Ecuador; 9 Scripps Institute of Oceanography, University of California San Diego, San Diego, California, United States of America; University of Sydney, AUSTRALIA

## Abstract

The kelp forests of southern South America are some of the least disturbed on the planet. The remoteness of this region has, until recently, spared it from many of the direct anthropogenic stressors that have negatively affected these ecosystems elsewhere. Re-surveys of 11 locations at the easternmost extent of Tierra del Fuego originally conducted in 1973 showed no significant differences in the densities of adult and juvenile *Macrocystis pyrifera* kelp or kelp holdfast diameter between the two survey periods. Additionally, sea urchin assemblage structure at the same sites were not significantly different between the two time periods, with the dominant species *Loxechinus albus* accounting for 66.3% of total sea urchin abundance in 2018 and 61.1% in 1973. Time series of Landsat imagery of the region from 1998 to 2018 showed no long-term trends in kelp canopy over the past 20 years. However, ~ 4-year oscillations in canopy fraction were observed and were strongly and negatively correlated with the NOAA Multivariate ENSO index and sea surface temperature. More extensive surveying in 2018 showed significant differences in benthic community structure between exposed and sheltered locations. Fish species endemic to the Magellanic Province accounted for 73% of all nearshore species observed and from 98–100% of the numerical abundance enumerated at sites. Fish assemblage structure varied significantly among locations and wave exposures. The recent creation of the Yaganes Marine Park is an important step in protecting this unique and biologically rich region; however, the nearshore waters of the region are currently not included in this protection. There is a general lack of information on changes in kelp forests over long time periods, making a global assessment difficult. A complete picture of how these ecosystems are responding to human pressures must also include remote locations and locations with little to no impact.

## Introduction

Giant kelp (*Macrocystis pyrifera*) forests are the foundation of marine communities on many shallow rocky coasts of the world’s cold-water marine habitats and constitute one of the most diverse and productive ecosystems on the planet [1–5]. Globally, there is concern for the future of kelps (Laminariales) as temperate nearshore systems are increasingly impacted by both local stressors such as sedimentation, pollution, overfishing and direct harvest as well as global stressors related to climate change such as marine heatwaves [5–11]. Marine heatwaves in particular are predicted to increase and have been shown to have dramatic negative effects on kelps [9,12]. Despite a plethora of recent scientific studies and popular media headlines showing dramatic losses of kelp forests, often marked by sharp transitions to low diversity urchin barrens or turf algae, a comprehensive global analysis showed that kelp loss is not uniform, but rather highly spatially variable in both magnitude and direction of change [10]. That global study concluded that local and regional-scale drivers can dominate kelp forest dynamics and noted that many regions of the world suffer a dearth of data on kelps, precluding our ability to understand changes that might occur in a rapidly warming ocean. The most thorough and longest time series come from very few regions of the world [10,11], which limited our understanding of how these ecosystems functions and how best to conserve them.

The southern cone of South America is the confluence of water masses from three great oceans (Pacific, Atlantic, and Southern oceans), with a mix of species of temperate and sub-Antarctic distributions that creates a unique area of marine endemism with high biodiversity value [13,14]. During the voyage of the Beagle, Darwin noted the lush kelp forests of Tierra del Fuego and the high diversity of species found within them [15]. Despite being the most southern kelp forests in the world, few ecological studies of this important ecosystem have been conducted. The region is described as having dense *M*. *pyrifera* forests with few sea urchins [16–18]. Unlike other regions of the world, the *M*. *pyrifera* beds in southern Chile do not appear to be controlled by sea urchins; however, sea urchins and the gastropod *Tegula atra* have been shown to influence kelp recruitment dynamics in lower latitudes within the region [19–21]. In addition to *M*. *pyrifera*, understories of other kelp taxa (e.g., *Lessonia*) are also noted to be important to the ecology and ecosystem function of southern Chile [3,18,22].

The southern tip of South America consists of largely unfragmented ecosystems, with relatively low anthropogenic impacts, and very low human population density [23]. The Mitre Peninsula (MP) is the easternmost extent of Isla Grande de Tierra del Fuego, with Isla de los Estados (IE) lying 29 km to the east and separated by Le Maire Strait. IE has a long history of occupation dating back at least two millennia by the Yaghan people before its "rediscovery" by the Dutch navigators Schouten and Le Maire in 1616 while looking for an alternative passage to the Strait of Magellan [24]. Dangerous seas with strong waves, winds, and currents, along with persistent fog made the island and its surroundings rocky islets a graveyard for early sailing ships [25]. As a result of these hazardous conditions, the Argentine government built an ill-fated lighthouse in 1884, which became the inspiration for Jules Vernes’s adventure novel “The Lighthouse at the End of the World” [26]. IE is a provincial reserve for “ecology, history, and tourism” with access limited to tours from Ushuaia, Argentina. The only settlement at IE is the Puerto Parry Naval Station, located in a deep and narrow fjord on the northern coast of the island.

Geographical isolation and hazardous sea conditions have limited the number of scientific studies and, therefore, our understanding of this remote region. Various explorers navigated the region after its discovery, but no permanent settlement was established until 1829. In 1882, the Argentine Geographic Institute commissioned the first official scientific expedition to IE, with much of the scientific work in the 19^th^ and early 20^th^ century related to mapping and meteorology of the region. In 1973, The United States National Science Foundation research vessel RV Hero surveyed the kelp communities around IE and the MP [18]. This seminal work was one of the first to quantitatively describe the kelp forest communities of the southern cone of South America and established a baseline for this little-known region.

Here we provide the first long-term examination of kelp forest communities in this very remote and understudied region, which adds to our understanding of how these ecosystems function in a location with limited direct human impacts. The objectives of our work were to: (1) compare kelp community metrics in 2018 with the 1973 kelp forest surveys of IE and MP, (2) compare current benthic communities and fish assemblages between IE and MP, (3) examine the utility of satellite imagery for observing kelp at IE and MP and develop a time series of kelp canopy for the region, and (4) discuss variability and synchrony in kelp canopy dynamics and its relationship to environmental drivers.

## Methods

### Ethics statement

Data were collected by all authors in a collaborative effort. Non-invasive research was conducted, which included photographs and visual estimates described in the methods below. The Republic of Argentina granted all necessary permission to conduct this research. No vertebrate sampling was conducted and therefore no approval was required by the University of Hawaii Institutional Animal Care and Use Committee. Our data are publicly available at Data Dryad: doi.org/10.5061/dryad.6djh9w0xd

### Site description

**Fig 1:**
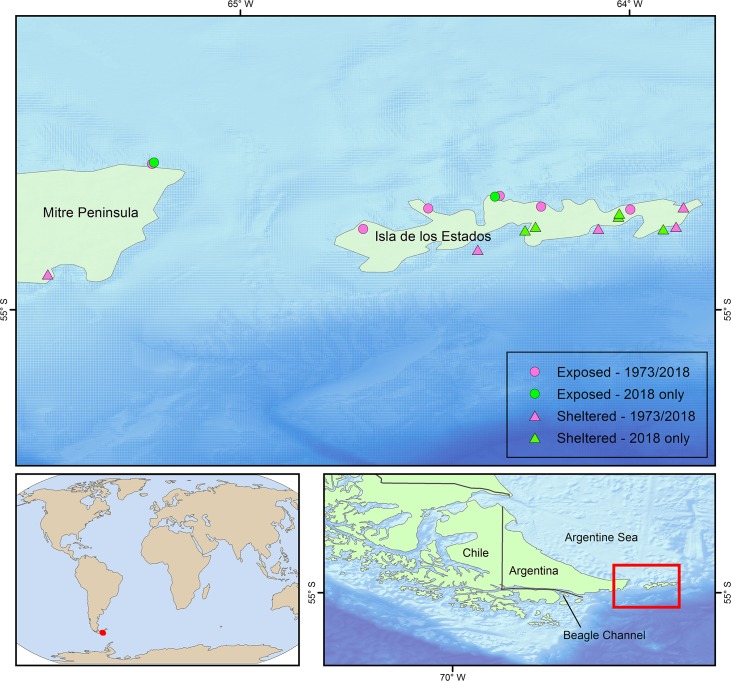
Sampling locations around Isla de los Estados and Mitre Peninsula. Red–locations sampled in 1973 and 2018; green– 2018 sampling only. Circles represent exposed locations; triangles represent sheltered locations. Data from GEBCO Compilation Group (2019) GEBCO 2019 Grid (doi:10.5285/836f016a-33be-6ddc-e053-6c86abc0788e) and Natural Earth, free vector and raster map data @ naturalearthdata.com.

The MP and IE are the terminus of the South American continent and the 7,200 km long Andes mountain range [27,28]. MP is the easternmost part of Isla Grande de Tierra del Fuego and is bordered to the south by the Beagle Channel and to the north by the Argentine Sea. IE is ~ 65 km long east-west, and 15 km wide with a total land area of 534 km^2^ (Fig 1). It is deeply indented by numerous bays with the highest peak at 823 m. The climate of the island is strongly influenced by the subpolar low-pressure system, with strong winds from the west and > 200 cm of rainfall annually [29].

### Giant kelp canopy biomass

We analyzed 312 Landsat images (across the 5 TM, 7 ETM+, and 8 OLI sensors) to produce site-based and regional *M*. *pyrifera* canopy fraction time series for the region. Atmospherically-corrected imagery were obtained from the United States Geological Survey (earthexplorer.usgs.gov). Emergent canopy density was estimated using Multiple Endmember Spectral Mixing Analysis [30]. This procedure models each pixel as a linear combination of one static kelp canopy spectral endmember and one of 30 seawater spectral endmembers unique to each image. The use of dynamic seawater endmembers accounts for changing water conditions (e.g. phytoplankton blooms, suspended sediments, sunglint) between image dates. The fractional cover of kelp canopy within each pixel is determined from the model with the lowest root mean squared error.

Kelp canopy fraction has been shown to be linearly correlated to canopy biomass density at sites in California [31,32] and this canopy pixel fraction to biomass relationship has been applied to *M*. *pyrifera* sites at several locations across the Americas (Baja California, SE Alaska, Cape Horn [33,34]). The vast majority of available Landsat imagery in the region was acquired starting in 1998, likely due to data storage limitations from this remote area prior to that date. This analysis, therefore, covers the 20-year period between 1998–2018. Landsat imagery was only available between the months of August–April, as usable imagery is unobtainable in high latitudes during winter months due to low sunlight levels. To validate kelp canopy fraction retrievals to the in-situ *M*. *pyrifera* SCUBA survey data, we used the mean kelp canopy fraction from the four Landsat pixels surrounding each *in situ* survey location (Jan–Mar 2018). This method is similar to that used for ground truthing kelp canopy in California [32].

In order to compare *in situ M*. *pyrifera* density estimates and Landsat imagery, we used a reduced major axis regression (Matlab code lsqfitgm). We *a priori* removed two sites due to low geographic precision, and three sites owing to a lack of kelp pixels within 500 m of the survey locations. These last three sites all occurred on the south side of land features, which tend to cast shadows and decrease reflectance at high southern latitudes, making satellite observations of kelp canopy difficult.

Time series of summed kelp canopy density were produced for each of the remaining 18 sites for the time period 1998–2018. These time series represent summed kelp canopy density for all pixels within 100 m of the site coordinates (a 400 m range was used for two sites, and a 1000-m range was used for two other sites due to the presence of few (< 5) kelp pixels within 100 m). Dates where > 25% of pixels at a site were obscured by clouds were removed from the analysis. The time series of summed canopy density for each site were then interpolated onto trimesters (average of 1.55 valid observations of each site for a trimester). In order to compare the kelp canopy dynamics across sites, we standardized each time series by dividing all values by the maximum summed canopy density observed at the site across the entire 20-year period, termed proportional kelp canopy density. This effectively accounted for differences in available kelp habitat (rocky reef area) between sites in order to focus on the relative dynamics. Regional estimates of kelp canopy dynamics were generated in a similar fashion from all observable kelp canopy pixels in MP and IE. The mean kelp canopy density across all images within a trimester was calculated in order to account for variations due to tides and currents. We evaluated long term trends in the trimesterly and annual regional data using a generalized least squares regression (nlme package in R, [35]), with a corAR1 correlation structure to quantify and account for temporal autocorrelation.

Regional-scale drivers of kelp canopy density, based on the NOAA Multivariate ENSO Index (MEI; www.esrl.noaa.gov/psd/enso/mei/) and sea surface temperature near MP and IE (SST; NOAA Optimum Interpolation Sea Surface Temperature; www.ncdc.noaa.gov/oisst) were compared to both site and regional-scale kelp canopy dynamics using Pearson’s correlation coefficients from simple linear correlations. In order to examine the effect of these potential drivers on interannual kelp canopy dynamics we applied a 2-year running mean to the site and regional-scale kelp canopy time series as well as the SST and MEI time series. To understand how these drivers were related to one another, MEI was compared to fluctuations in SST. The relationship between temperature and seawater nitrate concentration was fit using a generalized additive model (mgcv package in R, [36]). Seawater temperature and nitrate concentrations at MP in 2006–2007 were taken from previously published work [37].

### In-situ surveys: Invertebrates and fishes

During the 1973 U.S. National Science Foundation surveys around IE and the southernmost coast of Tierra del Fuego, scuba divers quantified sea urchin and *M*. *pyrifera* (juveniles and adults) densities (number of individuals^.^m^-2^) over 50 m^2^ areas using either a 1-m^2^ quadrat along a 50 m transect or a 2-m^2^ quadrat along a 25 m transect, depending on sea conditions [18]. Transects were laid along isobaths in order to control for depth effects. Adult *M*. *pyrifera* were defined arbitrarily as plants with fronds reaching the surface or with four or more stipes. *M*. *pyrifera* holdfast diameters were measured to the nearest cm as an indication of plant size and age. In 1973, size distributions for the dominant sea urchin in the region were obtained. Field notes from the 1973 expedition were transcribed and entered into a database.

In 2018, characterization of the benthos was conducted by scuba divers along two 25-m long transects at the same sampling location as the 1973 surveys. Survey sites were relocated based on coordinates, nautical charts, and detailed descriptions of each site from Dayton’s digitized field notes. Transects were run parallel to the shoreline, with target depths as close as possible to the 1973 surveys. For sessile and mobile invertebrates, the number of individuals was estimated on 1-m of either side of the transect line (50 m^2^). For colonial organisms (sponges, some cnidarians, bryozoans, and some tunicates) colonies, rather than individuals, were counted. Only non-cryptic invertebrates ≥ 1 cm were enumerated. A second diver counted the number of kelp (*M*. *pyrifera* and *Lessonia* spp.) stipes within 1-m on either side of the transect. *M*. *pyrifera* holdfast diameters were measured by a third diver as an indication of plant size and age [18]. In 2018, additional stations were surveyed for a total of 18 stations and 36 transects. To examine the epifauna on *M*. *pyrifera*, we placed an entire plant in a large plastic bag, removed the holdfast, and brought the bag to the surface.

For fish surveys, a scuba diver counted and sized all fishes within 1-m of either side of a 25 m transect line (50 m^2^) at each survey site. The transect extended to the surface or as far as visibility allowed, including species associated with the kelp canopy and water column. Total fish lengths were estimated to the nearest cm. In addition, photographs were taken *in situ* to assist with species identification, document underwater coloration, and associated habitat. Fish species biogeographic affinities and trophic group designations were obtained from published literature [38–40]

### Statistical analyses

Comparisons of adult and juvenile *M*. *pyrifera* stipe counts and kelp holdfast diameter between 1973 and 2018 at the same locations were conducted using paired t-tests. Sea urchin assemblage structure between 1973 and 2018 was compared using multivariate analysis of variance (MANOVA). The multivariate test statistic Pillai’s Trace was used because it is robust to heterogeneity of variance and is less likely to involve type I errors than are comparable tests [41].

Benthic community characteristics included the following metrics: 1) total species: S—the number of species in each sample (i.e. species with non-zero counts), 2) species richness (Margalef): d = (S-1)/Log(N) where S is the total number of species present and N is the total number of individuals, 3) Shannon-Weiner diversity: H´ = -Ʃ p_i_ln(p_i_), where *p*_*i*_ is the proportion of all individuals counted that were of taxa *I*, 4) Pielou's evenness: J = H´/ln(S), where S is the total number of species present. All values in parentheses are one standard deviation of the mean unless otherwise noted.

Student’s t-tests were used to compare kelp plant and stipe densities between species, locations, and exposures. Drivers of benthic and fish assemblage structure were investigated using permutation-based multivariate analysis of variance (PERMANOVA). A Bray–Curtis similarity matrix was created from abundance of benthic taxa and fish species from transect data. Permutation of residuals under a reduced model (Sums of squares Type III–partial) with 999 permutations was used in these analyses. Locations (MP and IE) and exposures (exposed and sheltered) were treated as a fixed factor in two-way PERMANOVAs with interactions. Exposures were assigned subjectively based on the orientation of the location to the predominant swell direction (south and east = exposed, north = sheltered). Prior to analysis, benthic taxa and fish species were ln(x+1) transformed. Principal Coordinate Analysis (PCO) was used to display benthic and fish assemblage structure between locations and exposures in ordination space. The primary taxa vectors driving the ordination (Pearson correlation Product movement correlations ≥ 0.5) were overlaid on the PCO plots to visualize the major taxa that explained the spatial distribution patterns observed. All PERMANOVAs and PCOs were conducted using Primer v6.

## Results

### Kelp canopy time series

There was a strong, positive relationship between the mean pixel kelp canopy fraction and the *in-situ* density of adult *M*. *pyrifera* in the survey area (r^2^ = 0.763, p = 0.005), suggesting that the Landsat method worked well in the region for estimating kelp canopy density. Time series of canopy fraction for each of the 18 sites for the time period 1998–2018 showed much variability through time at all sites with a strong seasonal pattern and interannual variation in maximum canopy density, but no strong trend over time (S1 Fig). We found that there was no evidence for a long-term trend in kelp canopy fraction (p = 0.581). When we conducted this analysis on an annual basis there was also no evidence for a long-term canopy trend (p = 0.857).

There were differences in interannual variability between sites, as some sites displayed extensive, dense canopies while other sites lacked canopy in a given year. There appears to be an oscillatory pattern across the sites where there is a sizable amount of kelp canopy for ~4 years followed by a period with little kelp canopy (S2 Fig). This synchrony between sites is evidence for a low frequency, regional scale driver of kelp canopy density.

**Fig 2:**
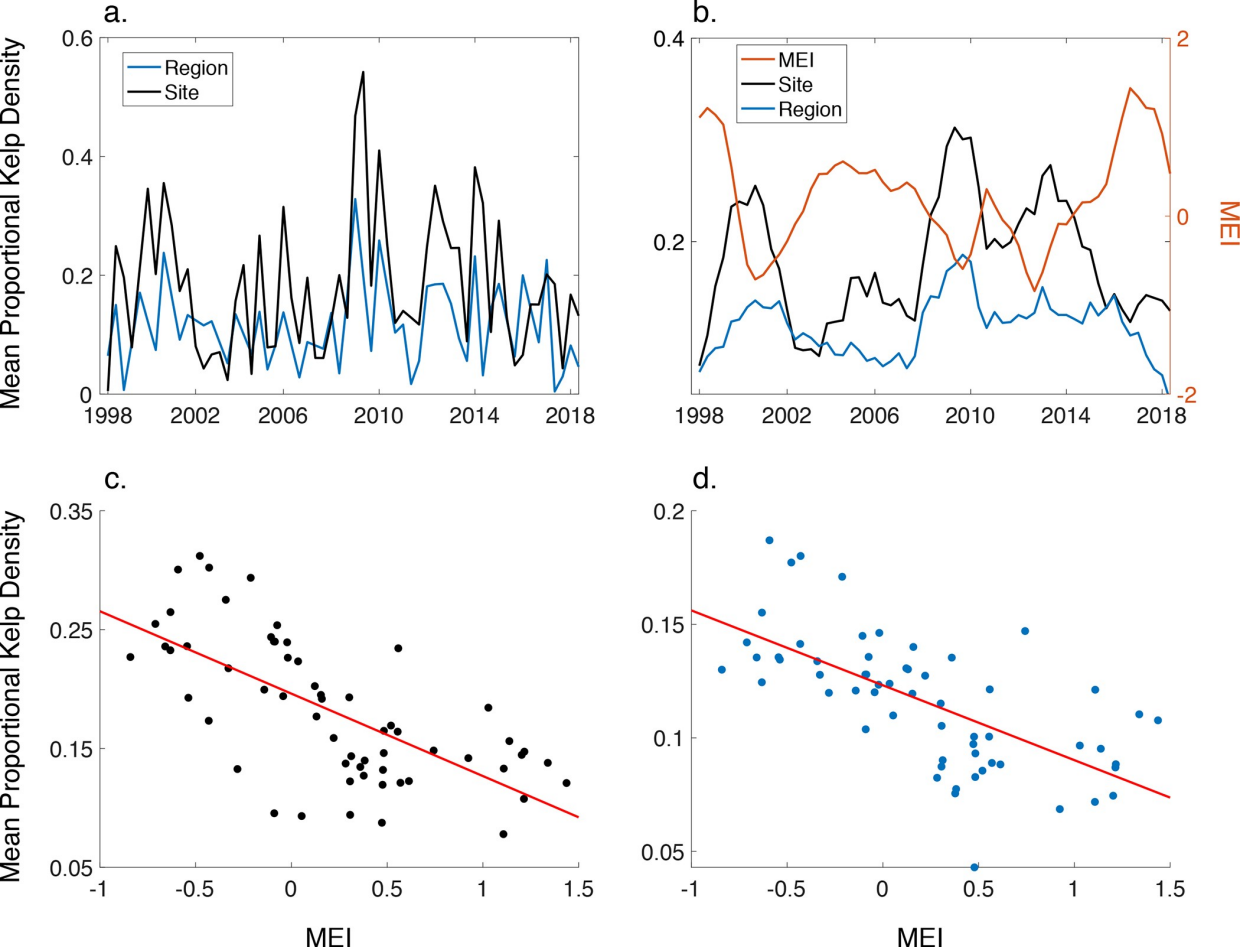
Kelp canopy density from Landsat imagery. a) Mean proportional kelp canopy density at the trimester time scale for both site (black) and regional (blue) scales. b) Mean proportional kelp canopy density with a 2-year running mean at both the site (black) and regional (blue) scales along with the 2-year running mean of the NOAA Multivariate ENSO Index (MEI), lagged by one year. c) Relationship between the site scale mean proportional kelp canopy density and MEI shown in b. Red line shows the linear fit between the variables. d) Relationship between the regional scale mean proportional kelp canopy density and MEI shown in b. Red line shows the linear fit between the variables.

We investigated this low frequency synchrony among sites by examining the entire regional kelp canopy dataset. While there was a strong seasonal cycle evident at the regional scale (Fig 2A), long-term trends (2-year running mean) showed a similar pattern to those seen in the 18 sites (r = 0.777, p < 0.001; Fig 2B). Furthermore, there was a negative relationship to the MEI at both the site scale (r = -0.671, p < 0.001; Fig 2C) and regional scale (r = -0.655, p < 0.001; Fig 2D) with a one-year lag. Monthly means of SST were significantly and positively correlated with the MEI when MEI was lagged by one year (r = 0.702, p < 0.001). Proportional kelp density was significantly and negatively related to the 2-year running means of monthly SST, with a one-year lag at both the site (r = -0.786, p < 0.001) and regional scale (r = -0.702, p < 0.001). There was a significant and negative relationship between SST and seawater nitrate concentrations using seawater samples taken in the TdF region (r^2^ = 0.64, p < 0.001), with very little nitrate found in seawater when temperatures exceeded 8^o^ C.

### Historical *in situ* comparisons of benthic communities

**Fig 3:**
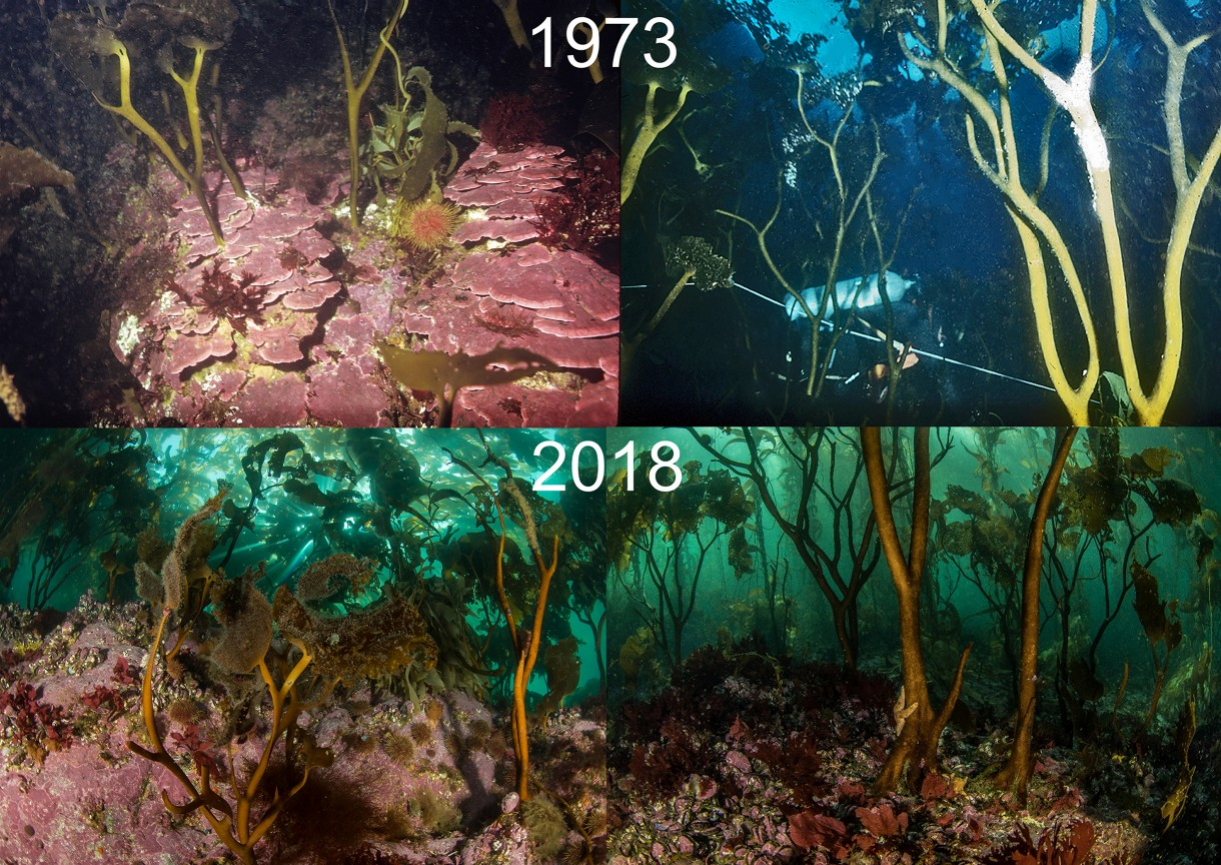
*Lessonia vadosa* at Isla de los Estados, 1973 and 2018.

Densities of adult *M*. *pyrifera* plants (no^.^m^-2^) at the same survey sites were not significantly different between 1973 ($\bar{X}$ = 0.442 ± 0.529) and 2018 ($\bar{X}$ = 0.590 ± 0.7518; t = 0.685, p = 0.265). Similarly, densities of juvenile *M*. *pyrifera* plants at the same survey sites were not significantly different between 1973 ($\bar{X}$ = 0.83 ± 1.65) and 2018 ($\bar{X}$ = 0.67 ± 0.70; t = 0.369, p = 0.654). Average kelp holdfast diameter was also similar between 1973 and 2018 at the same sites ($\bar{X}$ = 33.28 ± 11.46 cm and $\bar{X}$ = 43.12 ± 11.18 cm, respectively; t = 0.142, p = 0.164). While *Lessonia* spp. were not enumerated in 1973, descriptions and photographs show a similar distribution between the two time periods, with *L*. *vadosa* most common shoreward of the *M*. *pyrifera* belt and *L*. *flavicans* predominately found seaward of the *M*. *pyrifera* belt (Fig 3).

Sea urchin assemblage structure was not significantly different between the two time periods at the same locations (Pillai’s trace F_1,17_ = 0.724, p = 0.552) with the dominant species *Loxechinus albus* accounting for 63.1% of total sea urchin abundance in 1973 and 66.6% in 2018. *Pseudechinus magellanicus* was the next most abundant sea urchin in both time periods (1973 = 23.8%; 2018 = 18.6%), followed by *Austrocidaris canaliculatum* (1973 = 6.9%; 2018 = 12.9%), and *Arbacia dufresnii* (1973 = 6.4%; 2018 = 1.9%). Densities of *L*. *albus* were not significantly different (t = 0.66, p = 0.51) between 1973 ($\bar{X}$ = 1.9 ± 2.3 individuals^.^m^-2^) and 2018 ($\bar{X}$ = 1.4 ± 1.9 individuals^.^m^-2^).

Sea stars (Asteroidea) are taxonomically diverse and at the time of the 1973 surveys, little was known about the taxonomy of this group within this region. As a result, only 9 taxa were described during the 1973 surveys compared with 22 in 2018, thus making comparisons between the two time periods difficult. The most abundant sea star during both time periods was *Anasterias antarctica*, which accounted for 65.3% of the total asteroid abundance in 1973 and 42.0% in 2018. *Cosmasterias lurida* was the second most common asteroid in 1973 (18.4% of the total), while it ranked third in 2018 comprising 14.5% of total sea star abundance during that time period. *Asterina fimbriata* ranked second in 2018 (19.3%) and third in 1973 (7.4%).

### Benthic communities

Overall density of *Lessonia* spp. plants ($\bar{X}$ = 1.07 ± 1.18) was statistically indistinguishable from the density of *M*. *pyrifera* plants ($\bar{X}$ = 0.94 ± 0.80; t = 0.53, p = 0.60). Adult *M*. *pyrifera* stipe density counts (no^.^m^-2^) were not significantly different between MP ($\bar{X}$ = 6.35 ± 2.11) and IE ($\bar{X}$ = 5.92 ± 2.96; t = 0.39, p = 0.71). However, density of adult *M*. *pyrifera* stipes was significantly higher at exposed stations at IE ($\bar{X}$ = 7.57 ± 2.76) compared to sheltered stations ($\bar{X}$ = 4.81 ± 2.61, t = 2.73, p = 0.01). Overall density of *L*. *flavicans* ($\bar{X}$ = 0.77 ± 1.07) was significantly higher than *L*. *vadosa* ($\bar{X}$ = 0.30 ± 0.30; t = 2.25, p = 0.03) and is a result of our transect locations, which had an average depth of 13.7 m (± 4.1 sd). *L*. *flavicans* was not found on transects at MP and *L*. *vadosa* was not found on transects at exposed sites. At protected stations at IE, density of *L*. *flavicans* ($\bar{X}$ = 1.20 ± 1.13) was significantly higher than *L*. *vadosa* ($\bar{X}$ = 0.50 ± 0.83, t = 2.11, p = 0.04)

We enumerated a total of 158 different benthic taxa from 13 phyla and 24 classes during underwater quantitative surveys of MP and IE (SOM 3). Demospongiae had the highest taxonomic richness (n = 25), followed by Gastropoda (n = 25), Asteroidea (n = 22), and Malacostraceae (n = 12). The average number of taxa observed on transects was similar between IE (44.9 ± 11.3) and MP (44.7 ± 7.5) (Table 1). However, numerical abundance was more than double at IE (23.2 ± 15.8) compared to MP (10.7 ± 5.7), which was driven primarily by the delicate barnacle *Balanus laevis*, which had a density 27 times higher at IE (7.3 ± 8.1) compared with MP (0.3 ± 0.2). Other community metrics were all higher at MP compared to IE (richness by 21%, diversity by 23%, and evenness by 17%).

**Table 1:** Benthic community characteristics based on 25 x 2 m transects (50 m^2^). IE–Isla de los Estados, MP–Mitre Peninsula. Values are means with standard deviations in parentheses from surveys in 2018.

Community metrics/Location	IE	MP	Total
Number of taxa	44.93 (11.31)	44.67 (7.51)	44.89 (10.58)
Number of individuals^.^m^-2^	23.20 (15.79)	10.67 (5.69)	21.11 (15.23)
Species richness (Margalef’s d)	16.02 (4.72)	19.46 (2.43)	16.59 (4.56)
Diversity (H')	2.24 (0.61)	2.70 (0.35)	2.32 (0.60)
Evenness (J')	0.59 (0.16)	0.71 (0.07)	0.61 (0.15)

Active suspension feeders accounted for 69.2% of the total benthic numerical abundance of invertebrates at IE and 45.1% at MP. The major taxa in this feeding group included the barnacle *Balanus laevis* (42.7%), unidentified encrusting Lepraliomorpha bryozoan (11.2%), and the bivalve mollusk *Gaimardia trapesina* (9.9%, Table 2). Herbivorous browsers accounted for 22.2% of the numerical abundance of benthic taxa at MP and 11.6% at IE. The Chilean sea urchin *Loxechinus albus* comprised 49.4% of the feeding group, followed by the smooth pink gastropod *Margarella violacea* (13.0%), and the small pink urchin *Pseudechinus magellanicus* (13.0%). Passive suspension feeders were the next most important feeding group at MP (21.4%) but relatively less abundant at IE (6.5%). The octamerous anemone *Bunodactis octoradiata* accounted for 58.4% of the abundance in this feeding group, followed by the white stinging anemone *Antothoe chilensis* (22.4%). Carnivores had similar relative contributions to total abundance of benthic taxa (MP = 8.4% and IE = 7.7%), with the Cinderella sea star *Anasterias antarctica* (18.9%) and the common hermit crab *Pagurus comptus* (18.5%) being the most important contributors to this feeding group. Deposit feeders accounted for 3.9% of total benthic abundance at IE and 2.9% at MP. The red spotted worm sea cucumber *Chiridota pisanii* (75.3%) and the orange brittlestar *Ophiomyxa vivipara* (17.5%) were the most important species in this feeding group.

**Table 2:** Top ten invertebrate taxa based on Index of Relative Dominance (IRD). IRD = % numerical abundance (number of individuals^.^m^-2^) x % frequency of occurrence (freq.). Feed. = feeding groups: 1 = passive suspension feeders, 2 –active suspension feeders, 3 = herbivorous browsers, 4 = carnivores, 5 = omnivores, 6 = deposit feeders.

Superorder/ Order/Infraorder	Name	Feed.	Num^.^m^-2^ (sd)	% num m^-2^	% freq.	IRD
Sessilia	*Balanus laevis*	2	6.07 (7.81)	12.20	77.78	949
Echinidea	*Loxechinus albus*	3	1.33 (1.60)	2.67	94.44	252
Lepraliomorpha	Unid encrust. bryozoan	2	1.59 (4.71)	3.20	72.22	231
Actiniaria	*Bunodactis octoradiata*	1	0.95 (1.05)	1.91	83.33	159
Aplousobranchia	*Didemnum studeri*	2	0.74 (1.06)	1.48	88.89	132
Aplousobranchia	*Sycozoa gaimardi*	2	0.59 (1.51)	1.19	83.33	100
Aplousobranchia	*Polyzoa opuntia*	2	0.45 (0.55)	0.89	88.89	80
Imparidentia	*Gaimardia trapesina*	2	1.41 (4.79)	2.83	27.78	79
Forcipulatida	*Anasterias antarctica*	4	0.31 (0.35)	0.63	100.00	63
Temnopleuridea	*Pseudechinus magellanicus*	3	0.35 (0.62)	0.70	88.89	62

**Fig 4:**
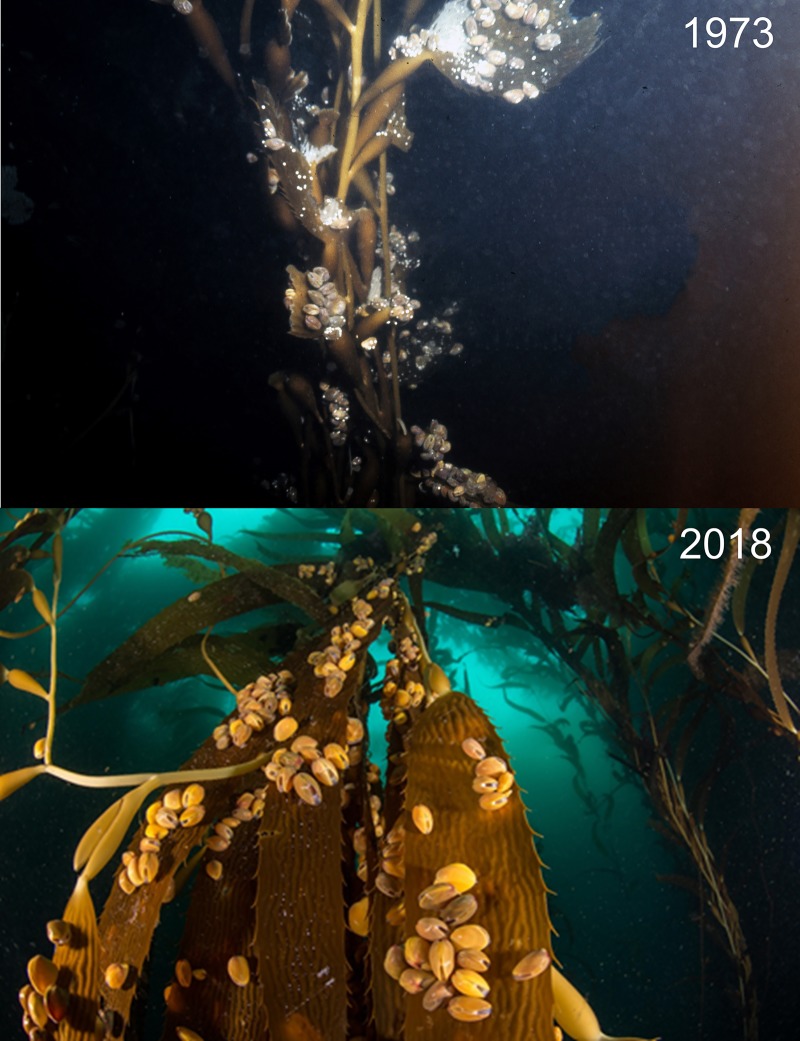
The bivalve mollusk *Gaimardia trapesina* growing on the fronds of *Macrocystis pyrifera*.

We harvested one large *M*. *pyrifera* plant (fronds and stipes = 49.1 kg; holdfast = 3.5 kg) and enumerated all individuals to the lowest possible taxa (N = 37, SOM 4). Several species of small amphipods (*Paramoera* sp., *Gondogeneia* sp., *Jassa* cf. *alonsoae*), isopods (*Ischyromene* cf. *eatoni*, *Neastacilla* sp.), and one free-living polychaete (*Proceraea cornuta*) accounted for ~ 95% of the individuals observed (Table 3). The bivalve *G*. *trapesina* and the bryozoan *Membranipora isabelleana* were observed growing on the fronds of *M*. *pyrifera* and at some locations, we observed entire kelp plants sunk by the weight of large aggregations of *G*. *trapesina* on the fronds (Fig 4). On the substrate, these bivalves were observed being consumed by several species of Asteroidea.

**Table 3:** Top ten taxa observed on single *M*. *pyrifera* plant. Fronds and stipes = 49.1 kg; holdfast = 3.5 kg. Percentages based on total number of non-colonial individuals (N = 18,770).

Phylum	Class	Subclass/Order/Superorder	Suborder/Family	Taxa	% total
Arthropoda	Malacostraca	Amphipoda	Pontogeneiidae	*Paramoera* sp.1	28.31
Arthropoda	Malacostraca	Amphipoda	Pontogeneiidae	*Gondogeneia* sp.1	28.08
Arthropoda	Malacostraca	Amphipoda	Ischyroceridae	*Jassa* cf. *alonsoae*	16.61
Annelida	Polychaeta	Phyllodocida	Syllidae	*Proceraea cornuta*	10.52
Arthropoda	Malacostraca	Amphipoda	Pontogeneiidae	Unidentified sp.	5.36
Arthropoda	Malacostraca	Isopoda	Sphaeromatidae	*Ischyromene* cf. *eatoni*	4.34
Arthropoda	Malacostraca	Amphipoda	Pontogeneiidae	*Gondogeneia macrodon*	1.31
Mollusca	Bivalvia	Imparidentia	Gaimardiidae	*Gaimardia trapesina*	1.30
Arthropoda	Malacostraca	Isopoda	Arcturidae	*Neastacilla* sp.1	1.07
Arthropoda	Malacostraca	Amphipoda	Ampithoidae	*Sunamphitoe* cf. *femorata*	0.99

Large mobile macro-invertebrates consisted mainly of sea urchins (Echinoidea) and sea stars (Asteroidea). Four species of sea urchins were observed on quantitative transects, with the total density (t = 0.4, p = 0.71) and the proportional contribution of each species (*X*^2^ = 2.3, p = 0.50) not significantly different between the two areas. *Loxechinus albus* was by far the most abundant species, accounting for 76% of total sea urchin density at MP ($\bar{X}$ = 1.3 ± 1.0 individuals^.^m^-2^) and 68% at IE ($\bar{X}$ = 1.3 ± 1.7 individuals^.^m^-2^). *Pseudechinus magellanicus* accounted for an additional 15% of sea urchin density at MP and 19% at IE. Sea urchins were commonly observed in cracks in the rocks or between the holdfasts of *M*. *pyrifera* and were observed feeding on drift algae. In some protected bays, large densities of *L*. *albus* were observed feeding on *Lessonia* spp.

A total of 22 sea star taxa were observed; however, five species accounted for 89% of the total numerical abundance. Of these, *Anasterias antarctica* accounted for 42% of the total, followed by *Asterina fimbriata* with 19%, *Cosmasterias lurida* with 15%, *Henricia obesa* with 8% and *H*. *studeri* with 4%. The contribution of *A*. *antarctica* was nearly identical between locations (IE = 42%, MP = 43%). However, *A*. *fimbriata* was nearly seven times more abundant at IE ($\bar{X}$ = 0.16 ± 0.38) compared to MP ($\bar{X}$ = 0.02 ± 0.05), contributing 21% to total sea star abundance at the former and 5% at the latter. *C*. *lurida* had similar densities among locations but comprised 23% of total sea star abundance at MP ($\bar{X}$ = 0.11 ± 0.10) and 14% at IE ($\bar{X}$ = 0.11 ± 0.13).

**Fig 5:**
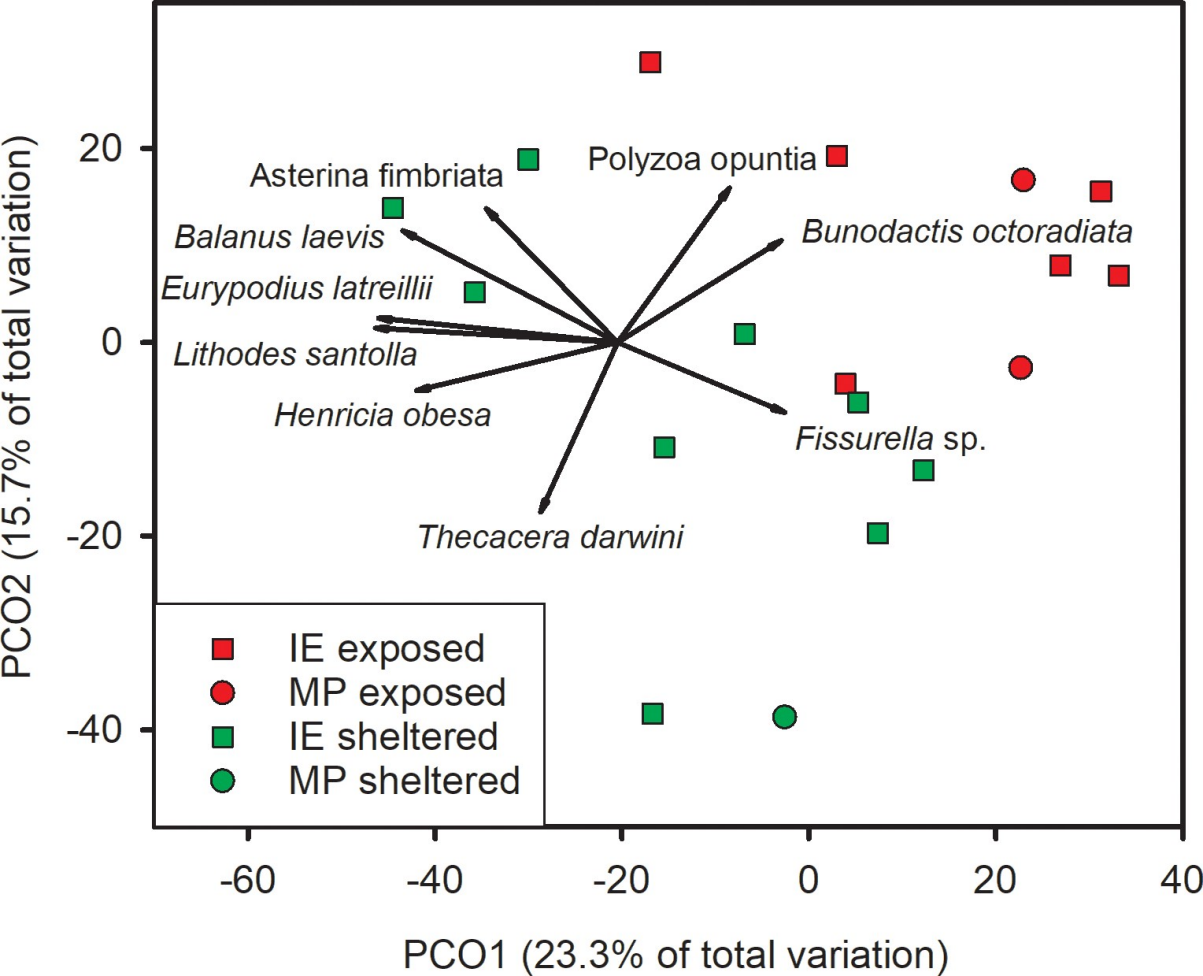
Principle coordinates analysis of benthic invertebrate community composition based on density (number of individuals^.^m^-2^) by location and exposure. Data were ln(x+1)-transformed prior to analyses. Vectors are the relative contribution and direction of influence of benthic components to the observed variation among sites (Pearson Product movement ≥ 0.5).

There was a significant difference in benthic community composition based on density (number of individuals^.^m^-2^) between exposures (i.e., exposed vs. sheltered) but not between locations or their interaction (Table 4). Exposed stations were more concordant in ordination space compared to sheltered locations and were most highly correlated with the abundances of the sea anemone *Bunodactis octoradiata* and the ascidian *Polyzoa opuntia* (Fig 5). Sheltered sites separated into two groups; one group was correlated with the limpet *Fissurella* sp. and the sea slug *Thecacera darwini*, while the other group was correlated with the Chilean king crab *Lithodes santolla*, camouflaged spider crab *Eurypodius latreillii*, the delicate barnacle *Balanus laevis*, the Cinderella sea star *Asterina fimbriata*, and the slender-armed sea star *Henricia obesa*.

**Table 4:** Comparison of benthic community composition based on density (number of individuals^.^m^-2^) between locations and exposures based on permutation-based multivariate analysis of variance (PERMANOVA).

Source	df	MS	Pseudo-F	P(perm)
Location	1	2420.4	1.184	0.279
Exposure	1	4523.1	2.212	0.006
Location x exposure	1	2316.3	1.133	0.314
Residuals	14	2045.2		
Total	17			

### Fishes

We conducted a total of 35 quantitative fish transects at 18 stations at IE (n = 32) and Mitre (n = 3), with an average depth of 13.7 m (± 4.1 sd). A total of 15 species of fishes from 7 families and 4 orders were observed on dives down to 25 m (Table 5). Of these 15 species, 13 were observed on quantitative transects, with the average size of individuals of all species combined only 9.9 cm TL (± 6.0). The Pink cuskeel *Genypterus blacodes* (n = 1, 38 cm TL), the Frogmouth *Cottoperca trigloides* (n = 4, 26.3 ± 7.5 cm TL) and the South American eelpout *Austrolycus depressiceps* (n = 2, 24.5 ± 12.0 cm TL) were the only three species larger than 20 cm TL observed on transects.

**Table 5:** Species of fishes observed during expedition at Isla de los Estados (IE) and Mitre Peninsula (MP). Pisc = piscivore; Inv = invertivore. Values are mean number of individuals^.^m^-2^, with one standard deviation of the mean in parentheses.–means the taxa was not observed. Family names in bold.

Family/ common name	Scientific name	Trophic group	IE	MP
**Agonidae**				
Armored fish	*Agonopsis chiloensis*	Inv	-	-
**Syngnathidae**				
Pipefish	*Leptonotus blainvilleanus*	Inv	-	-
**Ophidiiformes**				
Pink cuskeel	*Genypterus blacodes*	Pisc, Inv	-	0.3 (0.6)
**Bovichtidae**				
Frogmouth	Cottoperca trigloides*	Pisc, Inv	0.2 (0.4)	0.3 (0.6)
**Nototheniidae**				
Magellanic rock cod	*Paranotothenia magellanica*+	Inv	1.3 (2.8)	16.3 (13.4)
Rock cod	Patagonotothen brevicauda*	Inv	0.3 (0.7)	-
Rock cod	Patagonotothen cornucola*	Inv	7.5 (8.2)	-
Rock cod	Patagonotothen longipes*	Inv	0.4 (1.1)	-
Rock cod	Patagonotothen sima*	Inv	5.3 (3.2)	1.3 (2.3)
Rock cod	Patagonotothen squamiceps*	Inv	16.4 (15.8)	1.0 (1.7)
Rock cod	Patagonotothen tessellata*	Inv	5.5 (14.3)	-
**Harpagiferidae**				
Spiny plunder fish	Harpagifer bispinis*	Inv	0.6 (1.2)	-
**Zoarcidae**				
South American eelpout	Austrolycus depressiceps*	Pisc, Inv	0.1 (0.3)	0.3 (0.6)
Eelpout	Crossostomus chilensis*	Pisc, Inv	0.2 (0.6)	-
Eelpout	Pogonolycus marinae*	Pisc, Inv	0.1 (0.3)	0.3 (0.6)

* Magellanic endemic

^+^ Magellanic, Subantarctic Is. endemic

Nototheniidae was the most specious family, accounting for 47% of all species observed (Table 5). Species endemic to the Magellanic Province accounted for 73% of all nearshore species observed. These regional endemics accounted for 100% of the numerical abundance on quantitative surveys at IE and 98% at MP. All species had benthic affinities except for the bentho-pelagic Magellanic rock cod *Paranotothenia magellanica*. The majority of the fishes observed were invertebrate feeders, while three feed on fishes and invertebrates (e.g., Pink cuskeel, Frogmouth, South American eelpout).

Comparisons of fish assemblage structure was significantly different between locations, exposures, and their interaction (Table 6). Fish assemblage structure at exposed stations were significantly different between IE and MP (t = 3.93, p = 0.027) but not at sheltered stations (t = 1.33, p = 0.203). Fish assemblage structure at IE was significantly different between exposures (t = 1.62, p = 0.036) but not at MP (t = 5.27, p = 0.398).

**Table 6:** Comparison of fish assemblage composition based on density (number of individuals^.^m^-2^) between locations and exposures based on permutation-based multivariate analysis of variance (PERMANOVA).

Source	df	MS	Pseudo-F	P(perm)
Location	1	7600.6	4.411	0.007
Exposure	1	4535.8	2.633	0.041
Location x exposure	1	6655.1	3.863	0.004
Residuals	14	1722.9		
Total	17			
Pair-wise comparisons				
Exposure	t	P(perm)		
Sheltered	1.330	0.203		
Exposed	3.928	0.027		
Location	t	P(perm)		
IE	1.621	0.036		
MP	5.275	0.398		

**Fig 6:**
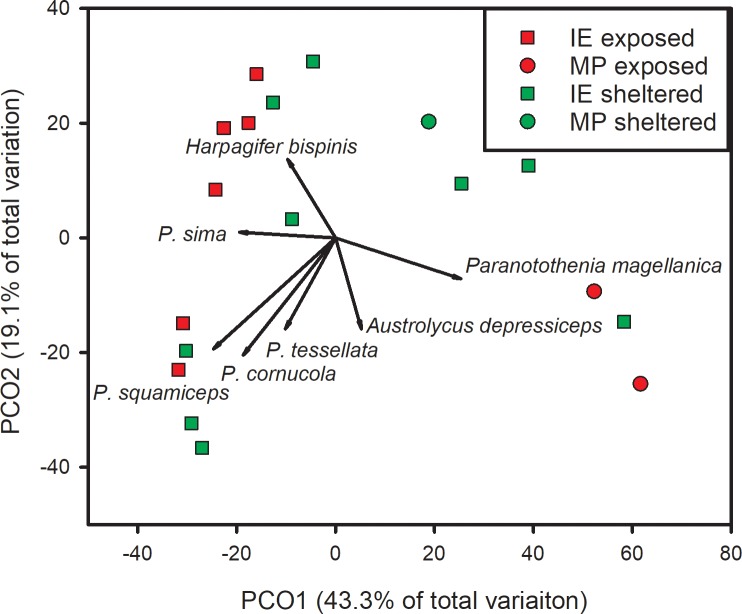
Principle coordinates analysis of fish assemblage composition based on density (number of individuals^.^m^-2^) by location. Data were ln(x+1)-transformed prior to analyses. Vectors are the relative contribution and direction of influence of fish assemblage components to the observed variation among sites (Pearson Product movement ≥ 0.5). *P*. = *Patagonotothen* spp.

PCO1 explained 43.3% of the total variation of fish assemblage structure in ordination space (Fig 6). MP exposed stations were situated at the upper end of this axis and IE stations were clustered more towards the lower end, with exposed stations having higher concordance compared to sheltered stations. *Patagonotothen sima*, *P*. *squamiceps*, and *Harpagifer bispinis* were most closely correlated with exposed IE stations, while *Paranotothenia magellanica* was most closely correlated with MP exposed stations.

## Discussion

The kelp forest communities of the southernmost tip of South America play a key role in structuring the entire ecosystem of the region but have not been well studied due to their remoteness and extreme weather conditions [16,18,42]. Here we used Landsat imagery and a rare, early survey and detailed field notes from 1973 to make comparisons in key aspects of kelp forest communities over a 20-year and a 45-year period, respectively. We found a remarkable degree of similarity in relative abundance of kelp, sea urchins, and seastars from the *in-situ* surveys conducted during the two time periods. We note that these were only two points in time, separated by 45 years. In addition, the small sample sizes, due to the logistics and remoteness of this area, makes it difficult to distinguish between a true negative effect and a false negative (Type 2 error). However, the consistency of our results and the concordance with the long-term satellite data, gives us confidence that the kelp forest communities within the region have not changed greatly since 1973.

Landsat imagery allowed a time series to be built and we were able to identify a few key drivers of kelp canopy variability over time (e.g., SST, nitrate concentrations). Based on satellite data, there is little evidence for long-term trends in kelp canopy over the past 20 years at most sites. Mean monthly SST was significantly and positively correlated with the MEI and this relationship may be due to the influence of ENSO on the Antarctic Circumpolar Wave, which produces alternating cool/warm water conditions as it circles the Antarctic continent [43,44]. Measurements of SST and nitrate concentrations taken in MP in 2006–2007 show a negative and nonlinear relationship [37]. The increase in nitrate (and possibly other nutrients) in the cooler water associated with low MEI values could lead to increased growth and recruitment of *M*. *pyrifera*, as has been observed in other systems [30]. Years with high MEI values tend to have extended periods of > 8°C seawater during the summer growing season, leading to extended periods of surface waters with little nitrate.

Satellite data in this region only became available in 1998, providing a 20-year time series. The decadal-scale cycles seen in this and other marine autotrophs, which tend to be associated with low frequency climate oscillations [30,45,46], require time series of many decades (often >40 years in coastal areas) before long term trends can be accurately assessed [32,47,48].

Bell and colleagues [49] conducted a robust analysis of 34 years of kelp canopy biomass estimates over multiple space and time scales off the coast of California. They found that there was a very slight increase in kelp canopy biomass over the length of the time series, but that much of the interannual variation could be explained by the North Pacific Gyre Oscillation. We see similar relationships here, where much of the interannual variation is associated with the MEI.

Dayton [18] described the kelp forests around IE as large, covering areas from hectares to several km^2^ and dominated by *M*. *pyrifera* with an understory of *Lessonia vadosa* in shallow water shoreward of the *M*. *pyrifera* belt and *L*. *flavicans* seaward of this belt. He noted that sea urchins were typically found in crevices and on kelp holdfasts, where they consumed drift algae and exerted little grazing pressure on upright *M*. *pyrifera*, although sea urchins were observed feeding on *Lessonia* spp. Dayton also noted *M*. *pyrifera* fronds covered with the small bivalve *G*. *trapezina* and occasionally predatory asteroids, which had climbed onto the kelp to eat the bivalves. Our results and observations are very similar to those observed by Dayton in 1973, suggesting that these communities have been persistent over decades.

In 1973, Dayton documented relatively low densities of *Loxechinus albus* at IE and MP and hypothesized that the Circumpolar Westwind Drift resulted in low larval availability to the region [18]. Our data show similarly low densities of *L*. *albus* between the two time periods and recent surveys in the Cape Horn Archipelago recorded even lower densities of this species ($\bar{X}$ = 0.6 ± 1.3 individuals^.^m^-2^) [22], which is consistent with Dayton’s prediction [18].

Dayton [18] notes that sea urchins in this region were almost never observed overgrazing *M*. *pyrifera* plants and the two main mortality sources for *M*. *pyrifera* were entanglement with drift algae and the heavy settlement of bivalves on kelp fronds. In our 2018 survey, we also did not observe large aggregations or ‘barrens’ of urchins. This is contrary to many regions of the world that are experiencing transitions from kelp forests to urchin barrens (e.g. Tasmania, Eastern Australia, Norway, Northern California; [12,50,51]. In many regions, the loss of fish or invertebrate predators can result in urchin barren formation [52] but this is unlikely to explain the lack of barrens in southern Argentina as the fish community is made up of smaller species that are unlikely to control urchins through predation. Similarly, the lack of mid-water and canopy dwelling fishes may contribute to the very heavy invertebrate fouling loads that we observed on *M*. *pyrifera* fronds in southern Argentina. Microcarnivorous reef fishes may play an important role in giant kelp forest communities by preventing infestations of mesograzers, which in turn can negatively affect kelp growth [53]. Kelp forests in southern Argentina have few canopy fishes and their absence may contribute to the heavy settlement that have been observed on kelp fronds in the region.

Unlike many kelp forests elsewhere around the world, no large predators appear to regulate the kelp forests of southern South America. Sea lions and fur seals eat mostly squid and small pelagic fishes [54], and the local otter *Lontra felina* does not eat sea urchins, but rather crustaceans and fishes [55]. Asteroids are the main predators in this ecosystem, which are trophic generalists that feed on a taxonomically diverse range of species [56,57]. The herbivore-kelp dynamics in this southern region appears to differ from those in the northern hemisphere and the lack of strong top-down control of kelp forests in this region means that other drivers likely contribute to the function of these ecosystems, including physical and biotic factors such as wave action, interspecific competition, and substratum availability [58].

Another distinguishing feature of these southern Argentina kelp forests is that the fish assemblages in the region are not a conspicuous component of the community [39]. Although species richness is low, the nearshore fishes of the region form a distinct biogeographic unit with high endemism. Despite this low richness, the region is a present-day hotspot of fish species formation [59], and is dominated by the radiation of highly specialized and geographically restricted species (e.g. Nototheniidae), which have the fastest rates of speciation of any marine region on earth [60].

### Threats

A recent global analysis of changes in kelp abundances over the past half-century identified declines in 38% of ecoregions for which there are data, with increases in 27% of ecoregions, and no detectable change in 35% of ecoregions [22]. These spatially variable trajectories reflect regional differences in the drivers of changes in kelp abundance and highlight the dynamic nature of kelp populations. For a limited number of sites with time series > 20 years, declines in kelp cover of > 60% have been documented [10,61]. The lack of information and data from many regions throughout the world hinders understanding the vulnerability of these foundational species to climate change and other anthropogenic stressors.

Climate-mediated changes are occurring to kelp forests worldwide due to increases in temperature, outbreaks of sea urchin populations and overfishing, which can act synergistically to exacerbate kelp declines [62]. While the ocean temperatures at the southern cone of South America are predicted to warm more slowly than other regions of the world [62], changes in productivity, current patterns, and species distributions will likely affect this region in the future. We identified 22 different taxa of sea stars during our expedition and based on global analysis of marine biodiversity, the southern tip of South America hosts the highest richness for sea stars on Earth [63]. Mobile invertebrates are disproportionately susceptible to warming seas and given the ecological importance of these species to the region, particularly asteroids, the effects of climate change could have devastating effects to the entire ecosystem.

The southern king crab (*Lithodes santolla*) previously constituted an economically valuable nearshore fishery off the southern tip of South America; however, declining yields in the 1980s led to the establishment of a fishery for false king crab (*Paralomis granulosa*), which historically was considered bycatch and of lower value [64]. Additionally, intensive fisheries for sea urchins in Chile and elsewhere have had devastating ecological consequences to kelp forest communities [65,66]. While there are currently no large-scale fisheries for sea urchins in southern Tierra del Fuego, experiences from elsewhere in Chile and around the world should be a cautionary tale for the develop of a fishery in this region. Overharvesting of these species likely has important consequences for the trophic dynamics of the entire kelp ecosystem of the region. Salmon farming has expanded exponentially along the southern coast of Chile in recent years and there are proposals to establish new farms in the Beagle Channel, both in Argentina and Chile. A large-scale outbreak of an infectious salmon anemia virus between 2008 and 2010 around Chiloé Island in Chile is one of the reasons for this expansion to more isolated and cooler waters [67]. In addition, salmon farming in Chile now requires more area to increase production, which has also led to an expansion of farming into the southern region of the country. Salmon is in the process of invading nearly the entire Patagonia region, and this introduced predator constitutes a major threat to biodiversity to the area [68]. In addition, copious amounts of feces, unconsumed feed, and dead fish greatly increase the nutrient load associated with salmon farms and antibiotics, pesticides, and other pharmaceuticals used by the salmon industry threaten water quality [69,70]. Eutrophication of coastal waters from salmon farms and other coastal activities has been shown to have negative consequences on the productivity and diversity of adjacent marine communities in the region and elsewhere [71–73]. The expansion of this industry, with a long history of environmental impact in Chile represents a threat to the biodiversity and conservation of the entire ecosystem of the region.

Another potential threat to the region is the direct harvest of kelp. In Chile, *Lessonia* spp. and *Macrocystis pyrifera* are under strong and increasing direct exploitation, primarily for alginate production and as a source of feed for abalone [74]. Due to overexploitation of kelp species in central and northern Chile [10] and the increasing interest in kelp biomass as fuel, harvesting is now moving to the southern region of the country.

### Conservation

This region is one of the last global refuges for kelp forest ecosystems and supports large populations of seabirds, marine mammals and has high biodiversity value due to high endemism and unique community composition [75]. The waters around Tierra del Fuego and Isla de los Estadios are important spawning and nursery areas for fisheries species that are exploited elsewhere along the Argentine Sea [76]. There is therefore an urgent need to protect this region for its biodiversity values and the ecosystem services it provides.

Argentina recently created the Yaganes fully protected MPA (69,000 km^2^), adjacent to the Diego Ramírez-Paso Drake Marine Park of 140,000 km^2^, declared by Chile in 2018. Although both are administratively and politically separated, they protect the same ecosystem. However, neither of these MPAs include the highly biodiverse nearshore areas of the Cape Horn Archipelago and Isla de los Estados. The regional government of Tierra del Fuego, with support for local community groups, has proposed the creation of a marine and terrestrial protected area at the Mitre Peninsula and marine protection around Isla de los Estados. Creation of a marine protection network between the Mitre Peninsula, Isla de los Estados, Cape Horn and the two large offshore MPAs of Yaganes and Diego Ramírez-Paso Drake Marine Park in Chile, would maximize the benefits for the protection of the entire region. Both countries have the historical opportunity to create a large marine park as a permanent symbol of friendship, which would achieve enormous conservation benefits.

There is generally a lack of information on changes in kelp forests over long time periods, making a global assessment difficult. Many of the long-term comparisons that have been published to date are from populated regions where many other stressors also prevail. A complete picture of how these important ecosystems are responding to human pressures must also include remote locations with little human impact. Our work provides a rare follow up to one of the few papers studying the ecology of kelp forests in the far southern region of South America. Re-examine of this extremely remote region is incredibly valuable in this age of climate change and gives us a better understanding on how these ecosystems function in the absence of major direct human impacts.

## Supporting information

S1 Fig Time series of summed Landsat kelp canopy fractions for all 18 sites. The black arrows show periods of time when most sites displayed little canopy kelp.
(TIF)

S2 Fig Time series of summed Landsat kelp canopy fractions for all 18 sites surveyed by the divers. These sites consisted of all pixels within (at least) 100m of the survey location and are divided into 4- month trimesters.
(TIF)
