# Supplementary figures and images for: Kelp forests at the end of the earth: 45 years later
Source: PLoS One. 2020 Mar 11;15(3):e0229259. doi: 10.1371/journal.pone.0229259 (PMC7065750; doi:10.1371/journal.pone.0229259)

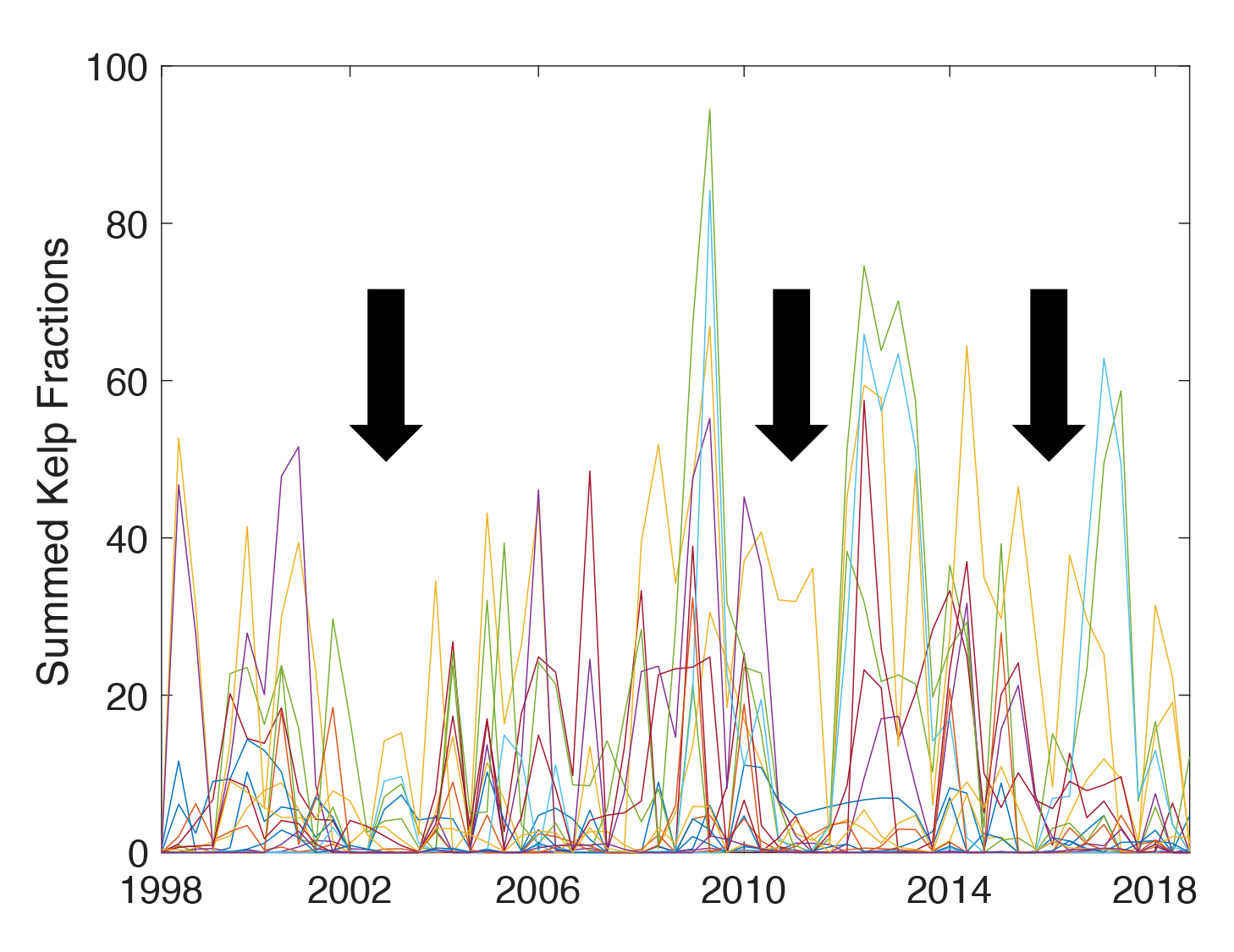

Supplement: S1 Fig — The black arrows show periods of time when most sites displayed little canopy kelp. (TIF) [file pone.0229259.s001.tif]

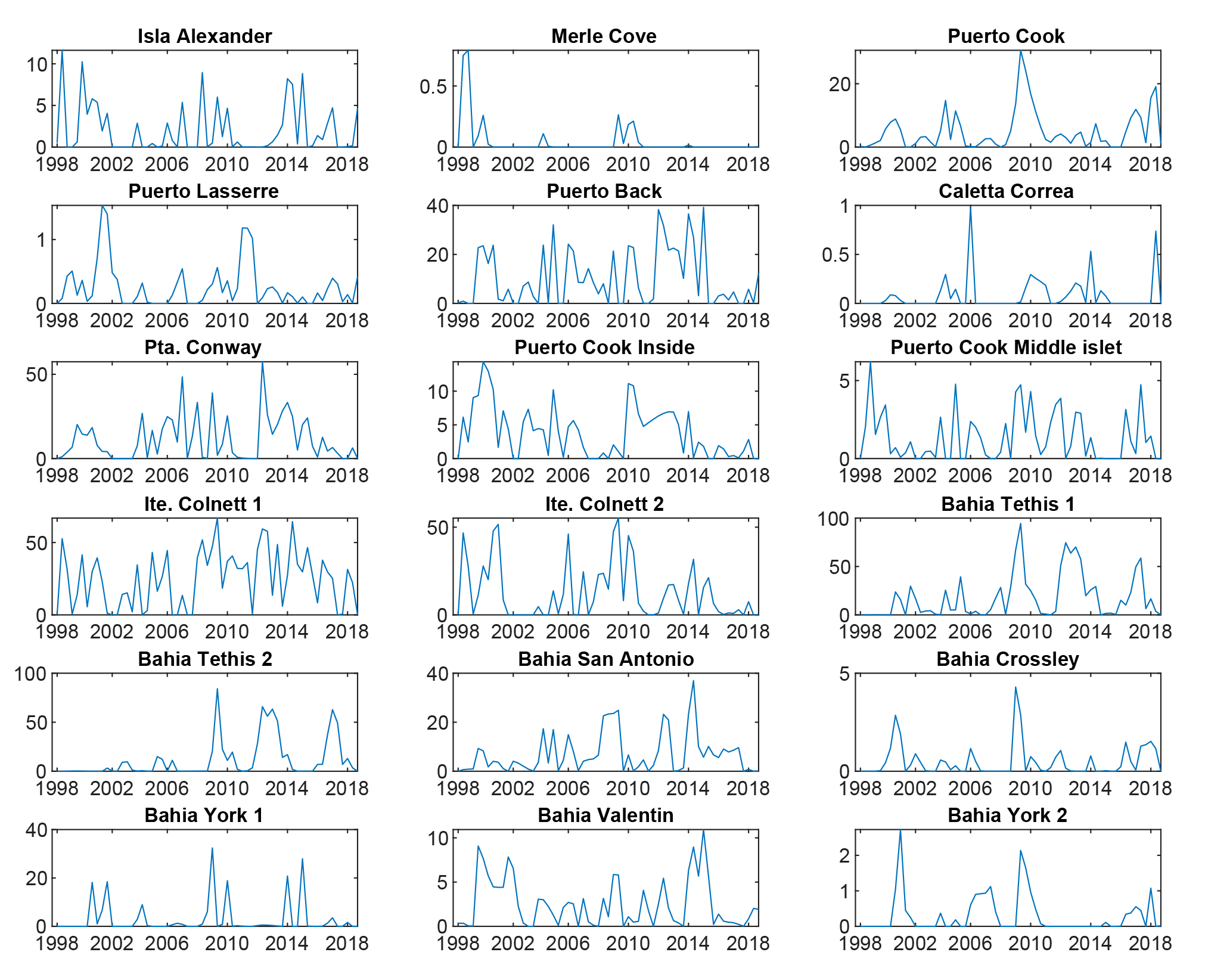

Supplement: S2 Fig — These sites consisted of all pixels within (at least) 100m of the survey location and are divided into 4- month trimesters. (TIF) [file pone.0229259.s002.tif]
